# Bis{2-[(guanidinoimino)­meth­yl]phenolato-κ^3^ *N*,*N*′,*O*}cobalt(III) chloride hemihydrate

**DOI:** 10.1107/S1600536813004534

**Published:** 2013-02-23

**Authors:** Elena A. Buvaylo, Vladimir N. Kokozay, Olga Yu. Vassilyeva, Brian W. Skelton

**Affiliations:** a Department of Inorganic Chemistry, Taras Shevchenko National University of Kyiv, 64/13 Volodymyrska Street, Kyiv 01601, Ukraine; b Centre for Microscopy, Characterisation and Analysis, University of Western Australia, 35 Stirling Highway, Crawley, WA 6009, Australia

## Abstract

The title compound, [Co(C_8_H_9_N_4_O)_2_]Cl·0.5H_2_O, is a solvatomorph of the corresponding trihydrate. Unlike in the structure of the latter compound, there are two different cations in the asymmetric unit of the title compound. The ligand mol­ecules are deprotonated at the phenol O atom and octa­hedrally coordinate the Co^III^ atoms through the azomethine N and phenolate O atoms in a *mer* configuration. In the crystal, the cations, chloride ions and lattice water mol­ecules are linked by N—H⋯O, N—H⋯Cl, O—H⋯Cl and O—H⋯O inter­actions, forming a two-dimensional network parallel to (10-1).

## Related literature

For direct synthesis using metal powders, see: Chygorin *et al.* (2012 ▶). For solvatomorphism, see: Desiraju (2004 ▶); Bernstein (2005 ▶); Nangia (2006 ▶); Brittain (2012 ▶). For the structure of the trihydrate solvatomorph of the title compound, see: Chumakov *et al.* (2006 ▶). For the structures of two different solvated crystalline forms of a related Schiff base ligand, see: Gutierrez *et al.* (2011 ▶).
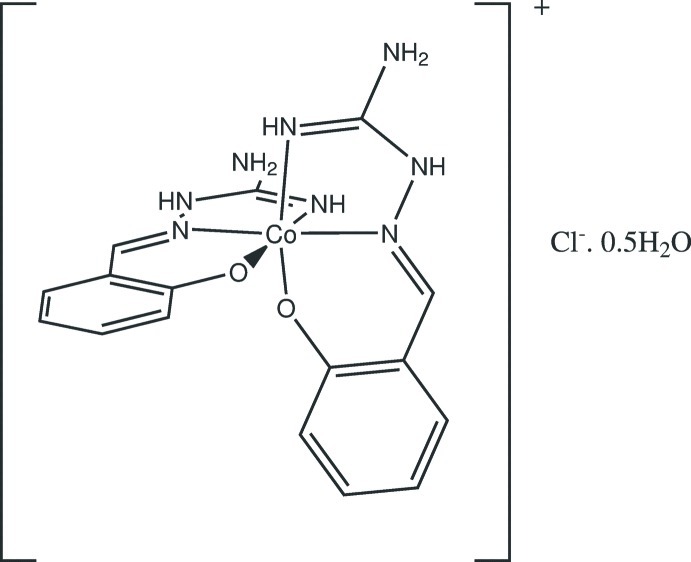


## Experimental

### 

#### Crystal data

[Co(C_8_H_9_N_4_O)_2_]Cl·0.5H_2_O
*M* *_r_* = 457.77
Triclinic, 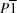

*a* = 9.9043 (2) Å
*b* = 10.2078 (2) Å
*c* = 18.5358 (4) Å
α = 100.773 (2)°
β = 92.019 (2)°
γ = 91.458 (2)°
*V* = 1838.84 (7) Å^3^
*Z* = 4
Mo *K*α radiation
μ = 1.11 mm^−1^
*T* = 100 K
0.39 × 0.31 × 0.17 mm

#### Data collection

Oxford Diffraction Xcalibur diffractometer
Absorption correction: analytical [*CrysAlis PRO* (Agilent, 2011 ▶), derived from an expression by Clark & Reid (1995 ▶)] *T*_min_ = 0.720, *T*_max_ = 0.864
84837 measured reflections
23430 independent reflections
19519 reflections with *I* > 2σ(*I*)
*R*_int_ = 0.035

#### Refinement

*R*[*F* ^2^ > 2σ(*F* ^2^)] = 0.041
*wR*(*F* ^2^) = 0.107
*S* = 1.06
23430 reflections
522 parameters
2 restraints
H atoms treated by a mixture of independent and constrained refinement
Δρ_max_ = 1.07 e Å^−3^
Δρ_min_ = −0.80 e Å^−3^

### 

Data collection: *CrysAlis PRO* (Agilent, 2011 ▶); cell refinement: *CrysAlis PRO*; data reduction: *CrysAlis PRO*; program(s) used to solve structure: *SIR92* (Altomare *et al.*, 1994 ▶); program(s) used to refine structure: *SHELXL97* (Sheldrick, 2008 ▶); molecular graphics: *ORTEPII* (Johnson, 1976 ▶); software used to prepare material for publication: *WinGX* (Farrugia, 2012 ▶).

## Supplementary Material

Crystal structure: contains datablock(s) I, global. DOI: 10.1107/S1600536813004534/wm2724sup1.cif

Structure factors: contains datablock(s) I. DOI: 10.1107/S1600536813004534/wm2724Isup2.hkl

Additional supplementary materials:  crystallographic information; 3D view; checkCIF report

## Figures and Tables

**Table 1:** Selected bond lengths (Å)

Co1—N125	1.8914 (9)
Co1—N122	1.8955 (8)
Co1—O11	1.8967 (8)
Co1—N222	1.8987 (9)
Co1—N225	1.9017 (9)
Co1—O21	1.9290 (8)
Co2—N322	1.8863 (8)
Co2—N422	1.8918 (8)
Co2—N425	1.8945 (9)
Co2—N325	1.9026 (9)
Co2—O31	1.9041 (8)
Co2—O41	1.9202 (7)

**Table 2:** Hydrogen-bond geometry (Å, °)

*D*—H⋯*A*	*D*—H	H⋯*A*	*D*⋯*A*	*D*—H⋯*A*
N123—H123⋯O21^i^	0.88	2.29	2.8823 (12)	124
N125—H125⋯Cl1	0.88	2.36	3.1152 (9)	144
N126—H12*B*⋯O21^i^	0.88	2.44	3.0709 (13)	129
N223—H223⋯Cl2^ii^	0.88	2.34	3.0948 (10)	144
N226—H22*A*⋯O1	0.88	1.95	2.8177 (14)	167
N226—H22*B*⋯Cl2^ii^	0.88	2.7	3.4131 (12)	138
N323—H323⋯O41^iii^	0.88	2.17	2.8311 (11)	131
N325—H325⋯Cl2	0.88	2.77	3.5086 (9)	142
N326—H32*A*⋯Cl2	0.88	2.59	3.3801 (10)	149
N326—H32*B*⋯O1^iv^	0.88	2.14	2.9861 (14)	162
N423—H423⋯Cl1^v^	0.88	2.31	3.0960 (9)	149
N425—H425⋯Cl2^vi^	0.88	2.77	3.3659 (10)	126
N426—H42*B*⋯Cl1^v^	0.88	2.48	3.2573 (10)	148
O1—H1*B*⋯Cl1^vii^	0.83 (2)	2.28 (2)	3.0538 (10)	155 (2)
O1—H1*A*⋯O31^viii^	0.86 (2)	2.23 (2)	3.0227 (12)	153 (2)
O1—H1*A*⋯O41^viii^	0.86 (2)	2.28 (2)	2.8568 (12)	125 (2)

Symmetry codes: (i) 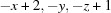; (ii) 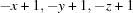; (iii) 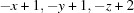; (iv) 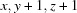; (v) 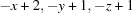; (vi) 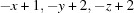; (vii) 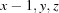; (viii) 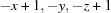.

**Fig. 1.**
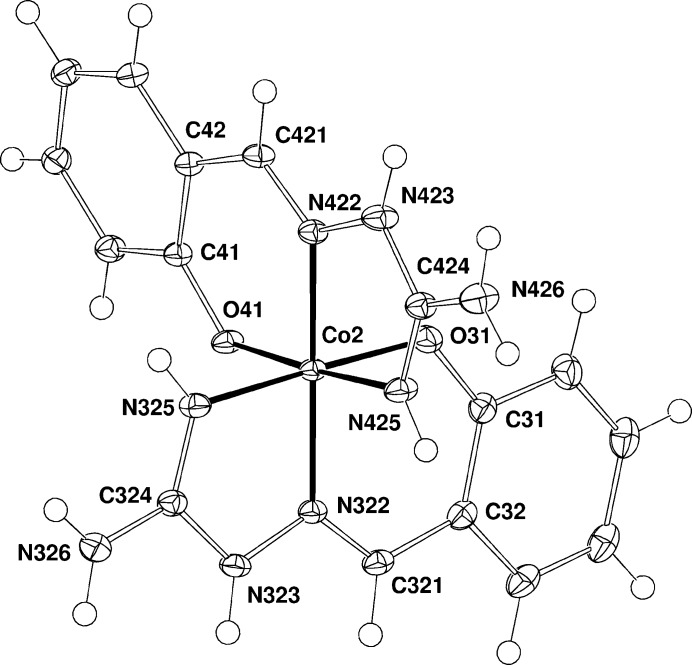
Molecular structure of one of the cations with the numbering scheme (the non-hydrogen atoms ellipsoids are shown at the 30% probability level).

**Fig. 2.**
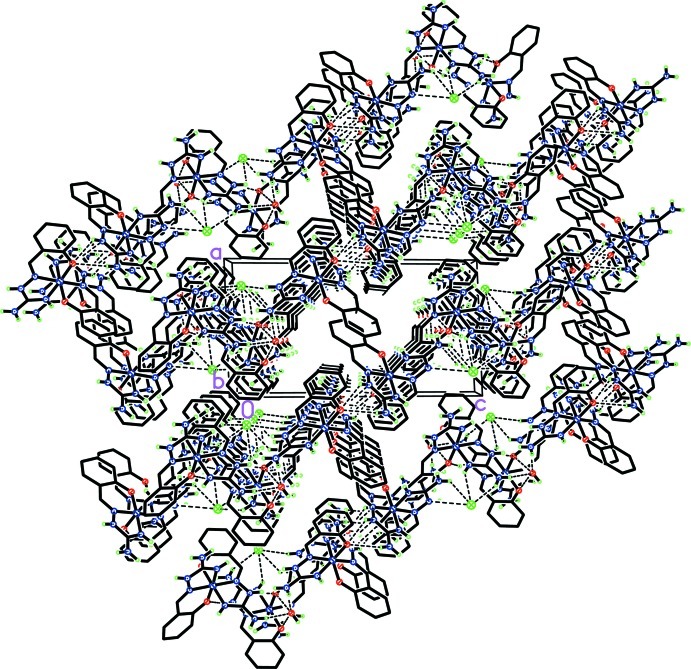
Perspective packing diagram viewed down the b axis revealing two-dimensional layers parallel to (101) formed by intermolecular N—H···O, N—H···Cl, O—H···Cl and O—H···O interactions (CH hydrogen atoms were omitted for clarity; hydrogen bonds shown as dashed lines).

## supplementary crystallographic information

### Comment

Solvatomorphism, sometimes called pseudopolymorphism, deals with crystals formed by the same substance but crystallized with different amounts or types of solvent molecules (Desiraju, 2004; Bernstein, 2005; Nangia, 2006; Brittain, 2012). The propensity of a given molecule towards hydrogen-bond formation with the solvent molecules leads to the formation of solvatomorphs of the parent compound with different packing motifs. Like polymorphism, solvatomorphism is commonly observed in structures of organic compounds and is of great significance in pharmaceuticals and materials.

The title compound is a solvatomorph of the complex bis(salicylideneguanylhydrazino-*N,N*',*O*)-cobalt(III) chloride trihydrate (refcode GEMJOY; Chumakov *et al.*, 2006). It was isolated in an attempt to prepare a heterometallic Co/Mn compound with the ligand, H*L*, that was synthesized from Schiff base formation of 2-hydroxybenzaldehyde with aminoguanidine hydrochloride. Details of the used synthetic approach as well as its applications were given by Chygorin *et al.* (2012). Remarkably, the related ligand, that was made from Schiff base formation of 2,6-dichloro-4-hydroxybenzaldehyde and aminoguanidine bicarbonate, iself was shown to form two solvated crystalline forms (Gutierrez *et al.*, 2011).

The title compound, [Co(C_8_H_9_N_4_O)_2_Cl]^.^0.5(H_2_O), is formed of discrete [Co*L*_2_]^+^ cations, chloride anions and water molecules of crystallizaion. Unlike GEMJOY there are two independent cations in the asymmetric unit of the title compound. Both cations are very similar and have no crystallographically imposed symmetry (Fig. 1). The ligand molecules are deprotonated at the phenol oxygen atom and coordinate to the Co^III^ atoms through the azomethine N and phenol O atoms in such a way that the Co^III^ atoms are octahedrally surrounded by two anionic ligands in a *mer* configuration. The Co–N/O distances (Table 1) fall in the range 1.8863 (8)–1.9290 (8) Å, the *trans* angles at the metal atoms are equal to 172.24 (4)–176.71 (4)°, the *cis* angles vary from 82.33 (4) to 94.86 (4)°. The coordination geometries around the Co^III^ atoms are similar to that found in GEMJOY (Chumakov *et al.*, 2006). The deprotonated ligand molecules adopt an almost planar conformation. In the crystal lattice, the cations, chloride ions, and lattice water molecules are linked together by intermolecular N—H···O, N—H···Cl, O—H···Cl and O—H···O interactions to form a two-dimensional network parallel to (101) (Fig. 2, Table 2).

### Experimental

Cobalt powder (0.03 g, 0.5 mmol), MnCl_2_^.^4H_2_O (0.10 g, 0.5 mmol), H*L*^.^HCl (0.21 g, 1 mmol) and methanol (30 ml) were heated to 323–333 K and magnetically stirred for 50 minutes. The resulting red-brown solution was filtered and allowed to stand at room temperature. Dark-red block-shaped microcrystals of the title compound were formed after 6 days. They were collected by filter-suction, washed with dry Pr^i^OH and finally dried *in vacuo* (yield: 25%).

### Refinement

H atoms were placed at idealized positions with a constrained C—H distance of 0.95 and an N—H distance of 0.88 Å and refined as part of riding models. *U*_iso_(H) values were set at 1.2*U*_eq_ of the attached atom. Water molecule H atoms were refined with geometries restrained to ideal values. The highest remaining electron density peaks (min, max) are 0.60 Å from Co1, and 0.18 Å from H323, respectively.

### Crystal data

**Table:**

[Co(C_8_H_9_N_4_O)_2_]Cl·0.5H_2_O	*Z* = 4
*M**_r_* = 457.77	*F*(000) = 940
Triclinic, *P*1	*D*_x_ = 1.654 Mg m^−^^3^
Hall symbol: -p 1	Mo *K*α radiation, λ = 0.71073 Å
*a* = 9.9043 (2) Å	Cell parameters from 29670 reflections
*b* = 10.2078 (2) Å	θ = 2.8–40.7°
*c* = 18.5358 (4) Å	µ = 1.11 mm^−^^1^
α = 100.773 (2)°	*T* = 100 K
β = 92.019 (2)°	Block, dark red
γ = 91.458 (2)°	0.39 × 0.31 × 0.17 mm
*V* = 1838.84 (7) Å^3^	

### Data collection

**Table:**

Oxford Diffraction Xcalibur diffractometer	23430 independent reflections
Graphite monochromator	19519 reflections with *I* > 2σ(*I*)
Detector resolution: 16.0009 pixels mm^-1^	*R*_int_ = 0.035
ω scans	θ_max_ = 40.5°, θ_min_ = 2.9°
Absorption correction: analytical [*CrysAlis PRO* (Agilent, 2011), derived from an expression by Clark & Reid (1995)]	*h* = −18→18
*T*_min_ = 0.720, *T*_max_ = 0.864	*k* = −18→18
84837 measured reflections	*l* = −33→33

### Refinement

**Table:**

Refinement on *F*^2^	Primary atom site location: structure-invariant direct methods
Least-squares matrix: full	Secondary atom site location: difference Fourier map
*R*[*F*^2^ > 2σ(*F*^2^)] = 0.041	Hydrogen site location: inferred from neighbouring sites
*wR*(*F*^2^) = 0.107	H atoms treated by a mixture of independent and constrained refinement
*S* = 1.06	*w* = 1/[σ^2^(*F*_o_^2^) + (0.0511*P*)^2^ + 0.4736*P*] where *P* = (*F*_o_^2^ + 2*F*_c_^2^)/3
23430 reflections	(Δ/σ)_max_ = 0.003
522 parameters	Δρ_max_ = 1.07 e Å^−^^3^
2 restraints	Δρ_min_ = −0.80 e Å^−^^3^

### Special details

**Table:**

Geometry. All s.u.'s (except the s.u. in the dihedral angle between two l.s. planes) are estimated using the full covariance matrix. The cell s.u.'s are taken into account individually in the estimation of s.u.'s in distances, angles and torsion angles; correlations between s.u.'s in cell parameters are only used when they are defined by crystal symmetry. An approximate (isotropic) treatment of cell s.u.'s is used for estimating s.u.'s involving l.s. planes.
Refinement. Refinement of *F*^2^ against ALL reflections. The weighted *R*-factor *wR* and goodness of fit *S* are based on *F*^2^, conventional *R*-factors *R* are based on *F*, with *F* set to zero for negative *F*^2^. The threshold expression of *F*^2^ > 2σ(*F*^2^) is used only for calculating *R*-factors(gt) *etc*. and is not relevant to the choice of reflections for refinement. *R*-factors based on *F*^2^ are statistically about twice as large as those based on *F*, and *R*- factors based on ALL data will be even larger.Water molecule hydrogen atoms were refined with geometries restrained to ideal values.

### Fractional atomic coordinates and isotropic or equivalent isotropic displacement parameters (Å^2^)

**Table:**

	*x*	*y*	*z*	*U*_iso_*/*U*_eq_	
Co1	0.827167 (13)	0.083852 (13)	0.376939 (7)	0.01187 (3)	
Co2	0.611810 (13)	0.650408 (13)	0.893594 (7)	0.01106 (3)	
Cl1	1.24863 (3)	−0.03691 (3)	0.287310 (15)	0.01975 (5)	
Cl2	0.19760 (3)	0.96175 (3)	0.937412 (15)	0.02252 (5)	
C11	0.61323 (10)	0.21108 (10)	0.46047 (5)	0.01394 (14)	
O11	0.68370 (9)	0.20168 (8)	0.40119 (4)	0.01830 (14)	
C12	0.62893 (10)	0.13118 (10)	0.51533 (5)	0.01361 (14)	
C121	0.71910 (10)	0.02208 (10)	0.50915 (5)	0.01423 (14)	
H121	0.7169	−0.0333	0.545	0.017*	
N122	0.80287 (9)	−0.00421 (8)	0.45719 (5)	0.01313 (12)	
N123	0.88511 (9)	−0.11340 (9)	0.45539 (5)	0.01570 (14)	
H123	0.8781	−0.1706	0.4853	0.019*	
C124	0.97792 (10)	−0.12184 (10)	0.40195 (6)	0.01488 (15)	
N125	0.96716 (9)	−0.03880 (9)	0.35708 (5)	0.01636 (14)	
H125	1.0209	−0.0395	0.3202	0.02*	
N126	1.06788 (10)	−0.21989 (10)	0.39836 (6)	0.01985 (16)	
H12A	1.1262	−0.2317	0.3632	0.024*	
H12B	1.0682	−0.272	0.4312	0.024*	
C13	0.55435 (11)	0.15794 (11)	0.57983 (6)	0.01724 (16)	
H13	0.5662	0.1041	0.6161	0.021*	
C14	0.46482 (11)	0.26027 (11)	0.59135 (6)	0.01894 (17)	
H14	0.4189	0.2803	0.636	0.023*	
C15	0.44326 (11)	0.33395 (11)	0.53561 (6)	0.01864 (17)	
H15	0.3791	0.4023	0.542	0.022*	
C16	0.51319 (11)	0.30936 (11)	0.47170 (6)	0.01784 (16)	
H16	0.494	0.3593	0.4343	0.021*	
C21	1.03067 (11)	0.29884 (10)	0.41005 (6)	0.01690 (16)	
O21	0.95157 (9)	0.21235 (8)	0.43595 (4)	0.01865 (14)	
C22	1.04017 (10)	0.30740 (10)	0.33508 (6)	0.01608 (15)	
C221	0.95235 (11)	0.23312 (10)	0.27791 (6)	0.01705 (16)	
H221	0.9631	0.2461	0.229	0.02*	
N222	0.85960 (9)	0.14963 (9)	0.28963 (5)	0.01468 (13)	
N223	0.77566 (11)	0.08933 (10)	0.23131 (5)	0.02135 (18)	
H223	0.774	0.1141	0.1883	0.026*	
C224	0.69561 (11)	−0.01190 (11)	0.24619 (6)	0.01745 (16)	
N225	0.70323 (9)	−0.03298 (9)	0.31335 (5)	0.01625 (14)	
H225	0.6551	−0.096	0.3281	0.02*	
N226	0.61571 (12)	−0.07642 (12)	0.19052 (6)	0.0265 (2)	
H22A	0.5602	−0.1413	0.1974	0.032*	
H22B	0.6186	−0.054	0.147	0.032*	
C23	1.13407 (11)	0.39789 (11)	0.31360 (8)	0.02135 (19)	
H23	1.141	0.4005	0.2629	0.026*	
C24	1.21588 (12)	0.48250 (13)	0.36462 (9)	0.0280 (3)	
H24	1.2803	0.5415	0.3496	0.034*	
C25	1.20178 (15)	0.47938 (13)	0.43866 (9)	0.0309 (3)	
H25	1.2545	0.5398	0.4746	0.037*	
C26	1.11214 (14)	0.38969 (12)	0.46114 (8)	0.0259 (2)	
H26	1.1055	0.3895	0.5121	0.031*	
C31	0.81815 (10)	0.45597 (10)	0.88106 (6)	0.01493 (15)	
O31	0.74001 (8)	0.52997 (8)	0.84714 (4)	0.01635 (12)	
C32	0.81293 (10)	0.44656 (10)	0.95638 (6)	0.01497 (15)	
C321	0.72252 (10)	0.52156 (10)	1.00603 (5)	0.01487 (15)	
H321	0.7267	0.5114	1.056	0.018*	
N322	0.63581 (8)	0.60210 (8)	0.98648 (4)	0.01268 (12)	
N323	0.55486 (9)	0.67200 (9)	1.03967 (5)	0.01466 (13)	
H323	0.5584	0.6633	1.086	0.018*	
C324	0.46931 (10)	0.75565 (10)	1.01209 (5)	0.01389 (14)	
N325	0.47942 (9)	0.76188 (9)	0.94286 (5)	0.01487 (13)	
H325	0.4291	0.8135	0.9207	0.018*	
N326	0.38727 (10)	0.82889 (10)	1.05904 (5)	0.01765 (15)	
H32A	0.3345	0.8869	1.0436	0.021*	
H32B	0.3864	0.8187	1.1051	0.021*	
C33	0.89567 (11)	0.35678 (11)	0.98563 (7)	0.01903 (17)	
H33	0.8917	0.352	1.0362	0.023*	
C34	0.98220 (11)	0.27588 (11)	0.94183 (7)	0.02107 (19)	
H34	1.0362	0.2146	0.9617	0.025*	
C35	0.98897 (11)	0.28567 (11)	0.86789 (7)	0.02109 (19)	
H35	1.0486	0.2307	0.8375	0.025*	
C36	0.91026 (11)	0.37418 (12)	0.83803 (7)	0.02006 (18)	
H36	0.9183	0.3801	0.7878	0.024*	
C41	0.39361 (9)	0.49962 (9)	0.80545 (5)	0.01243 (13)	
O41	0.47794 (8)	0.50957 (7)	0.86352 (4)	0.01390 (11)	
C42	0.39925 (10)	0.58283 (9)	0.75193 (5)	0.01252 (13)	
C421	0.50038 (10)	0.68781 (9)	0.75368 (5)	0.01374 (14)	
H421	0.5002	0.7356	0.7144	0.016*	
N422	0.59111 (9)	0.71972 (8)	0.80620 (4)	0.01260 (12)	
N423	0.68689 (10)	0.81840 (9)	0.80302 (5)	0.01686 (15)	
H423	0.6974	0.8534	0.7636	0.02*	
C424	0.76384 (10)	0.85724 (10)	0.86595 (5)	0.01469 (15)	
N425	0.74711 (9)	0.78781 (9)	0.91742 (5)	0.01589 (14)	
H425	0.7944	0.8033	0.9594	0.019*	
N426	0.85036 (11)	0.96201 (10)	0.86903 (6)	0.02079 (17)	
H42A	0.9025	0.9895	0.9087	0.025*	
H42B	0.8548	1.0031	0.8314	0.025*	
C43	0.30388 (10)	0.56265 (10)	0.69219 (5)	0.01464 (15)	
H43	0.3078	0.6194	0.6571	0.018*	
C44	0.20508 (10)	0.46259 (10)	0.68353 (6)	0.01570 (15)	
H44	0.141	0.4509	0.6433	0.019*	
C45	0.20096 (10)	0.37876 (10)	0.73500 (5)	0.01538 (15)	
H45	0.1347	0.3082	0.7291	0.018*	
C46	0.29211 (10)	0.39713 (10)	0.79446 (5)	0.01503 (15)	
H46	0.2864	0.3393	0.8289	0.018*	
O1	0.40782 (10)	−0.26387 (9)	0.20245 (5)	0.02312 (16)	
H1B	0.3487 (18)	−0.2225 (19)	0.2271 (11)	0.030 (5)*	
H1A	0.390 (2)	−0.3477 (16)	0.1956 (14)	0.048 (7)*	

### Atomic displacement parameters (Å^2^)

**Table:**

	*U*^11^	*U*^22^	*U*^33^	*U*^12^	*U*^13^	*U*^23^
Co1	0.01376 (5)	0.01180 (5)	0.01037 (5)	−0.00082 (4)	0.00077 (4)	0.00302 (4)
Co2	0.01325 (5)	0.01103 (5)	0.00952 (5)	−0.00078 (4)	−0.00045 (4)	0.00388 (4)
Cl1	0.02057 (10)	0.02147 (11)	0.01993 (10)	0.00328 (8)	0.00516 (8)	0.00978 (8)
Cl2	0.03053 (13)	0.02318 (11)	0.01366 (9)	0.00391 (10)	−0.00127 (9)	0.00303 (8)
C11	0.0155 (4)	0.0131 (3)	0.0139 (3)	0.0000 (3)	0.0011 (3)	0.0041 (3)
O11	0.0230 (3)	0.0180 (3)	0.0166 (3)	0.0059 (3)	0.0064 (3)	0.0085 (3)
C12	0.0144 (3)	0.0135 (3)	0.0136 (3)	0.0001 (3)	0.0012 (3)	0.0042 (3)
C121	0.0166 (4)	0.0148 (4)	0.0123 (3)	0.0003 (3)	0.0011 (3)	0.0051 (3)
N122	0.0149 (3)	0.0123 (3)	0.0126 (3)	0.0010 (2)	0.0002 (2)	0.0034 (2)
N123	0.0186 (3)	0.0143 (3)	0.0153 (3)	0.0035 (3)	0.0012 (3)	0.0051 (3)
C124	0.0143 (3)	0.0146 (4)	0.0148 (4)	0.0003 (3)	−0.0014 (3)	0.0007 (3)
N125	0.0154 (3)	0.0186 (4)	0.0155 (3)	0.0019 (3)	0.0029 (3)	0.0038 (3)
N126	0.0184 (4)	0.0184 (4)	0.0220 (4)	0.0053 (3)	−0.0013 (3)	0.0017 (3)
C13	0.0178 (4)	0.0198 (4)	0.0155 (4)	0.0016 (3)	0.0035 (3)	0.0062 (3)
C14	0.0188 (4)	0.0196 (4)	0.0196 (4)	0.0021 (3)	0.0058 (3)	0.0056 (3)
C15	0.0179 (4)	0.0172 (4)	0.0214 (4)	0.0030 (3)	0.0038 (3)	0.0044 (3)
C16	0.0196 (4)	0.0164 (4)	0.0191 (4)	0.0037 (3)	0.0020 (3)	0.0067 (3)
C21	0.0181 (4)	0.0128 (3)	0.0196 (4)	−0.0012 (3)	−0.0019 (3)	0.0032 (3)
O21	0.0250 (4)	0.0165 (3)	0.0138 (3)	−0.0065 (3)	−0.0014 (3)	0.0026 (2)
C22	0.0146 (4)	0.0137 (4)	0.0204 (4)	−0.0005 (3)	0.0020 (3)	0.0044 (3)
C221	0.0211 (4)	0.0157 (4)	0.0148 (4)	−0.0020 (3)	0.0042 (3)	0.0040 (3)
N222	0.0183 (3)	0.0145 (3)	0.0112 (3)	−0.0023 (3)	0.0007 (2)	0.0027 (2)
N223	0.0319 (5)	0.0207 (4)	0.0112 (3)	−0.0099 (4)	−0.0026 (3)	0.0043 (3)
C224	0.0204 (4)	0.0161 (4)	0.0156 (4)	−0.0029 (3)	−0.0032 (3)	0.0035 (3)
N225	0.0176 (3)	0.0164 (3)	0.0155 (3)	−0.0042 (3)	−0.0014 (3)	0.0058 (3)
N226	0.0330 (5)	0.0265 (5)	0.0191 (4)	−0.0122 (4)	−0.0104 (4)	0.0055 (4)
C23	0.0164 (4)	0.0168 (4)	0.0328 (6)	−0.0004 (3)	0.0048 (4)	0.0091 (4)
C24	0.0169 (4)	0.0191 (5)	0.0503 (8)	−0.0044 (4)	−0.0043 (5)	0.0143 (5)
C25	0.0296 (6)	0.0192 (5)	0.0440 (8)	−0.0086 (4)	−0.0168 (5)	0.0111 (5)
C26	0.0319 (6)	0.0173 (4)	0.0276 (5)	−0.0068 (4)	−0.0117 (5)	0.0054 (4)
C31	0.0136 (3)	0.0142 (4)	0.0177 (4)	−0.0008 (3)	0.0002 (3)	0.0052 (3)
O31	0.0179 (3)	0.0184 (3)	0.0144 (3)	0.0044 (2)	0.0024 (2)	0.0064 (2)
C32	0.0145 (3)	0.0140 (3)	0.0172 (4)	−0.0016 (3)	−0.0024 (3)	0.0057 (3)
C321	0.0168 (4)	0.0154 (4)	0.0133 (3)	−0.0017 (3)	−0.0024 (3)	0.0058 (3)
N322	0.0145 (3)	0.0133 (3)	0.0108 (3)	−0.0009 (2)	−0.0008 (2)	0.0040 (2)
N323	0.0176 (3)	0.0172 (3)	0.0100 (3)	−0.0001 (3)	0.0005 (2)	0.0047 (2)
C324	0.0152 (3)	0.0143 (3)	0.0119 (3)	−0.0018 (3)	−0.0008 (3)	0.0022 (3)
N325	0.0183 (3)	0.0155 (3)	0.0114 (3)	0.0021 (3)	−0.0003 (2)	0.0041 (2)
N326	0.0188 (4)	0.0198 (4)	0.0140 (3)	0.0015 (3)	0.0016 (3)	0.0018 (3)
C33	0.0181 (4)	0.0173 (4)	0.0227 (4)	−0.0001 (3)	−0.0048 (3)	0.0075 (3)
C34	0.0161 (4)	0.0174 (4)	0.0308 (5)	0.0006 (3)	−0.0045 (4)	0.0082 (4)
C35	0.0158 (4)	0.0179 (4)	0.0304 (5)	0.0020 (3)	0.0018 (4)	0.0063 (4)
C36	0.0180 (4)	0.0204 (4)	0.0235 (5)	0.0039 (3)	0.0041 (3)	0.0074 (4)
C41	0.0142 (3)	0.0126 (3)	0.0109 (3)	−0.0001 (3)	−0.0002 (3)	0.0034 (3)
O41	0.0169 (3)	0.0139 (3)	0.0115 (3)	−0.0035 (2)	−0.0028 (2)	0.0053 (2)
C42	0.0146 (3)	0.0125 (3)	0.0108 (3)	0.0004 (3)	−0.0005 (3)	0.0032 (3)
C421	0.0181 (4)	0.0129 (3)	0.0109 (3)	−0.0011 (3)	−0.0005 (3)	0.0042 (3)
N422	0.0154 (3)	0.0116 (3)	0.0114 (3)	−0.0018 (2)	0.0000 (2)	0.0040 (2)
N423	0.0221 (4)	0.0159 (3)	0.0133 (3)	−0.0075 (3)	−0.0023 (3)	0.0064 (3)
C424	0.0167 (4)	0.0143 (3)	0.0134 (3)	−0.0027 (3)	−0.0009 (3)	0.0039 (3)
N425	0.0184 (3)	0.0167 (3)	0.0133 (3)	−0.0045 (3)	−0.0032 (3)	0.0060 (3)
N426	0.0239 (4)	0.0202 (4)	0.0188 (4)	−0.0101 (3)	−0.0045 (3)	0.0077 (3)
C43	0.0173 (4)	0.0151 (4)	0.0119 (3)	0.0007 (3)	−0.0017 (3)	0.0040 (3)
C44	0.0154 (4)	0.0176 (4)	0.0137 (3)	0.0007 (3)	−0.0021 (3)	0.0024 (3)
C45	0.0148 (4)	0.0169 (4)	0.0140 (4)	−0.0017 (3)	−0.0002 (3)	0.0023 (3)
C46	0.0164 (4)	0.0155 (4)	0.0137 (3)	−0.0021 (3)	−0.0005 (3)	0.0047 (3)
O1	0.0218 (4)	0.0188 (4)	0.0273 (4)	−0.0004 (3)	0.0044 (3)	−0.0001 (3)

### Geometric parameters (Å, º)

**Table:**

Co1—N125	1.8914 (9)	C24—C25	1.391 (2)
Co1—N122	1.8955 (8)	C24—H24	0.95
Co1—O11	1.8967 (8)	C25—C26	1.3883 (19)
Co1—N222	1.8987 (9)	C25—H25	0.95
Co1—N225	1.9017 (9)	C26—H26	0.95
Co1—O21	1.9290 (8)	C31—O31	1.3183 (13)
Co2—N322	1.8863 (8)	C31—C36	1.4165 (15)
Co2—N422	1.8918 (8)	C31—C32	1.4199 (15)
Co2—N425	1.8945 (9)	C32—C33	1.4135 (14)
Co2—N325	1.9026 (9)	C32—C321	1.4367 (15)
Co2—O31	1.9041 (8)	C321—N322	1.2924 (13)
Co2—O41	1.9202 (7)	C321—H321	0.95
C11—O11	1.3122 (13)	N322—N323	1.3949 (12)
C11—C16	1.4208 (14)	N323—C324	1.3704 (13)
C11—C12	1.4244 (13)	N323—H323	0.88
C12—C13	1.4142 (14)	C324—N325	1.3038 (13)
C12—C121	1.4356 (14)	C324—N326	1.3468 (14)
C121—N122	1.2880 (13)	N325—H325	0.88
C121—H121	0.95	N326—H32A	0.88
N122—N123	1.3932 (12)	N326—H32B	0.88
N123—C124	1.3674 (14)	C33—C34	1.3820 (18)
N123—H123	0.88	C33—H33	0.95
C124—N125	1.2976 (14)	C34—C35	1.3965 (18)
C124—N126	1.3509 (14)	C34—H34	0.95
N125—H125	0.88	C35—C36	1.3866 (16)
N126—H12A	0.88	C35—H35	0.95
N126—H12B	0.88	C36—H36	0.95
C13—C14	1.3784 (16)	C41—O41	1.3257 (12)
C13—H13	0.95	C41—C46	1.4138 (14)
C14—C15	1.4007 (16)	C41—C42	1.4232 (13)
C14—H14	0.95	C42—C43	1.4114 (13)
C15—C16	1.3790 (16)	C42—C421	1.4432 (13)
C15—H15	0.95	C421—N422	1.2909 (13)
C16—H16	0.95	C421—H421	0.95
C21—O21	1.3318 (13)	N422—N423	1.3768 (12)
C21—C26	1.4099 (16)	N423—C424	1.3594 (13)
C21—C22	1.4148 (16)	N423—H423	0.88
C22—C23	1.4130 (15)	C424—N425	1.3030 (13)
C22—C221	1.4337 (15)	C424—N426	1.3448 (13)
C221—N222	1.2887 (13)	N425—H425	0.88
C221—H221	0.95	N426—H42A	0.88
N222—N223	1.3786 (13)	N426—H42B	0.88
N223—C224	1.3612 (14)	C43—C44	1.3796 (15)
N223—H223	0.88	C43—H43	0.95
C224—N225	1.3023 (14)	C44—C45	1.3965 (15)
C224—N226	1.3366 (14)	C44—H44	0.95
N225—H225	0.88	C45—C46	1.3817 (14)
N226—H22A	0.88	C45—H45	0.95
N226—H22B	0.88	C46—H46	0.95
C23—C24	1.3792 (19)	O1—H1B	0.831 (15)
C23—H23	0.95	O1—H1A	0.855 (16)
			
N125—Co1—N122	82.33 (4)	C224—N226—H22B	120
N125—Co1—O11	176.71 (4)	H22A—N226—H22B	120
N122—Co1—O11	94.41 (3)	C24—C23—C22	121.45 (12)
N125—Co1—N222	90.99 (4)	C24—C23—H23	119.3
N122—Co1—N222	172.24 (4)	C22—C23—H23	119.3
O11—Co1—N222	92.24 (4)	C23—C24—C25	118.35 (11)
N125—Co1—N225	91.40 (4)	C23—C24—H24	120.8
N122—Co1—N225	93.71 (4)	C25—C24—H24	120.8
O11—Co1—N225	88.41 (4)	C26—C25—C24	121.32 (12)
N222—Co1—N225	82.46 (4)	C26—C25—H25	119.3
N125—Co1—O21	90.54 (4)	C24—C25—H25	119.3
N122—Co1—O21	90.76 (4)	C25—C26—C21	121.52 (13)
O11—Co1—O21	89.88 (4)	C25—C26—H26	119.2
N222—Co1—O21	93.26 (4)	C21—C26—H26	119.2
N225—Co1—O21	175.32 (4)	O31—C31—C36	117.30 (10)
N322—Co2—N422	173.31 (4)	O31—C31—C32	125.34 (9)
N322—Co2—N425	91.61 (4)	C36—C31—C32	117.29 (9)
N422—Co2—N425	83.09 (4)	C31—O31—Co2	124.92 (7)
N322—Co2—N325	82.54 (4)	C33—C32—C31	120.31 (10)
N422—Co2—N325	93.51 (4)	C33—C32—C321	116.39 (9)
N425—Co2—N325	91.69 (4)	C31—C32—C321	123.26 (9)
N322—Co2—O31	94.86 (4)	N322—C321—C32	123.59 (9)
N422—Co2—O31	89.31 (3)	N322—C321—H321	118.2
N425—Co2—O31	90.92 (4)	C32—C321—H321	118.2
N325—Co2—O31	176.37 (4)	C321—N322—N323	118.73 (8)
N322—Co2—O41	91.23 (3)	C321—N322—Co2	127.74 (7)
N422—Co2—O41	94.22 (3)	N323—N322—Co2	113.34 (6)
N425—Co2—O41	176.60 (4)	C324—N323—N322	112.88 (8)
N325—Co2—O41	90.55 (4)	C324—N323—H323	123.6
O31—Co2—O41	86.96 (4)	N322—N323—H323	123.6
O11—C11—C16	117.83 (9)	N325—C324—N326	125.59 (10)
O11—C11—C12	125.45 (9)	N325—C324—N323	116.56 (9)
C16—C11—C12	116.72 (9)	N326—C324—N323	117.76 (9)
C11—O11—Co1	125.27 (7)	C324—N325—Co2	114.58 (7)
C13—C12—C11	120.15 (9)	C324—N325—H325	122.7
C13—C12—C121	116.76 (9)	Co2—N325—H325	122.7
C11—C12—C121	123.09 (9)	C324—N326—H32A	120
N122—C121—C12	123.01 (9)	C324—N326—H32B	120
N122—C121—H121	118.5	H32A—N326—H32B	120
C12—C121—H121	118.5	C34—C33—C32	121.06 (11)
C121—N122—N123	118.73 (8)	C34—C33—H33	119.5
C121—N122—Co1	128.28 (7)	C32—C33—H33	119.5
N123—N122—Co1	112.94 (6)	C33—C34—C35	118.93 (10)
C124—N123—N122	112.47 (8)	C33—C34—H34	120.5
C124—N123—H123	123.8	C35—C34—H34	120.5
N122—N123—H123	123.8	C36—C35—C34	121.18 (11)
N125—C124—N126	125.95 (10)	C36—C35—H35	119.4
N125—C124—N123	116.79 (9)	C34—C35—H35	119.4
N126—C124—N123	117.17 (10)	C35—C36—C31	121.20 (11)
C124—N125—Co1	114.90 (7)	C35—C36—H36	119.4
C124—N125—H125	122.6	C31—C36—H36	119.4
Co1—N125—H125	122.6	O41—C41—C46	118.06 (8)
C124—N126—H12A	120	O41—C41—C42	124.85 (8)
C124—N126—H12B	120	C46—C41—C42	117.07 (8)
H12A—N126—H12B	120	C41—O41—Co2	124.80 (6)
C14—C13—C12	121.56 (10)	C43—C42—C41	119.80 (9)
C14—C13—H13	119.2	C43—C42—C421	116.49 (8)
C12—C13—H13	119.2	C41—C42—C421	123.69 (8)
C13—C14—C15	118.39 (10)	N422—C421—C42	123.03 (8)
C13—C14—H14	120.8	N422—C421—H421	118.5
C15—C14—H14	120.8	C42—C421—H421	118.5
C16—C15—C14	121.43 (10)	C421—N422—N423	119.74 (8)
C16—C15—H15	119.3	C421—N422—Co2	128.41 (7)
C14—C15—H15	119.3	N423—N422—Co2	111.83 (6)
C15—C16—C11	121.47 (10)	C424—N423—N422	114.35 (8)
C15—C16—H16	119.3	C424—N423—H423	122.8
C11—C16—H16	119.3	N422—N423—H423	122.8
O21—C21—C26	117.74 (10)	N425—C424—N426	126.29 (9)
O21—C21—C22	125.39 (9)	N425—C424—N423	116.35 (9)
C26—C21—C22	116.87 (10)	N426—C424—N423	117.36 (9)
C21—O21—Co1	124.97 (7)	C424—N425—Co2	113.87 (7)
C23—C22—C21	120.36 (10)	C424—N425—H425	123.1
C23—C22—C221	116.38 (10)	Co2—N425—H425	123.1
C21—C22—C221	123.16 (9)	C424—N426—H42A	120
N222—C221—C22	123.24 (9)	C424—N426—H42B	120
N222—C221—H221	118.4	H42A—N426—H42B	120
C22—C221—H221	118.4	C44—C43—C42	121.65 (9)
C221—N222—N223	118.41 (9)	C44—C43—H43	119.2
C221—N222—Co1	128.88 (8)	C42—C43—H43	119.2
N223—N222—Co1	112.52 (6)	C43—C44—C45	118.77 (9)
C224—N223—N222	114.01 (9)	C43—C44—H44	120.6
C224—N223—H223	123	C45—C44—H44	120.6
N222—N223—H223	123	C46—C45—C44	120.81 (9)
N225—C224—N226	127.36 (10)	C46—C45—H45	119.6
N225—C224—N223	116.43 (9)	C44—C45—H45	119.6
N226—C224—N223	116.19 (10)	C45—C46—C41	121.87 (9)
C224—N225—Co1	114.34 (7)	C45—C46—H46	119.1
C224—N225—H225	122.8	C41—C46—H46	119.1
Co1—N225—H225	122.8	H1B—O1—H1A	110 (2)
C224—N226—H22A	120		

### Hydrogen-bond geometry (Å, º)

**Table:**

*D*—H···*A*	*D*—H	H···*A*	*D*···*A*	*D*—H···*A*
N123—H123···O21^i^	0.88	2.29	2.8823 (12)	124
N125—H125···Cl1	0.88	2.36	3.1152 (9)	144
N126—H12*B*···O21^i^	0.88	2.44	3.0709 (13)	129
N223—H223···Cl2^ii^	0.88	2.34	3.0948 (10)	144
N226—H22*A*···O1	0.88	1.95	2.8177 (14)	167
N226—H22*B*···Cl2^ii^	0.88	2.7	3.4131 (12)	138
N323—H323···O41^iii^	0.88	2.17	2.8311 (11)	131
N325—H325···Cl2	0.88	2.77	3.5086 (9)	142
N326—H32*A*···Cl2	0.88	2.59	3.3801 (10)	149
N326—H32*B*···O1^iv^	0.88	2.14	2.9861 (14)	162
N423—H423···Cl1^v^	0.88	2.31	3.0960 (9)	149
N425—H425···Cl2^vi^	0.88	2.77	3.3659 (10)	126
N426—H42*B*···Cl1^v^	0.88	2.48	3.2573 (10)	148
O1—H1*B*···Cl1^vii^	0.83 (2)	2.28 (2)	3.0538 (10)	155 (2)
O1—H1*A*···O31^viii^	0.86 (2)	2.23 (2)	3.0227 (12)	153 (2)
O1—H1*A*···O41^viii^	0.86 (2)	2.28 (2)	2.8568 (12)	125 (2)

Symmetry codes: (i) −*x*+2, −*y*, −*z*+1; (ii) −*x*+1, −*y*+1, −*z*+1; (iii) −*x*+1, −*y*+1, −*z*+2; (iv) *x*, *y*+1, *z*+1; (v) −*x*+2, −*y*+1, −*z*+1; (vi) −*x*+1, −*y*+2, −*z*+2; (vii) *x*−1, *y*, *z*; (viii) −*x*+1, −*y*, −*z*+1.
